# Nurse leaders' Attitudes, Self-Efficacy and training Needs for Implementing Evidence-Based Practice: Is It Time for a Change toward Safe Care?

**DOI:** 10.9734/BJMMR/2015/16487

**Published:** 2015-03-17

**Authors:** Jamileh Farokhzadian, Nahid Dehghan Nayeri, Fariba Borhani, Mohmmad Reza Zare

**Affiliations:** 1 Department of Community Health Nursing, School of Nursing and Midwifery, Kerman University of Medical Sciences, Kerman, Iran; 2 Nursing and Midwifery Care Research Center, School of Nursing and Midwifery, Tehran University of Medical Sciences, Tehran, Iran; 3 Department of Nursing Ethics, Medical Ethics and Law Research Center, Shahid Beheshti University of Medical Sciences, Tehran, Iran; 4 Afzalipour Hospital, University of Medical Sciences, Kerman, Iran

**Keywords:** Attitudes, self-efficacy, evidence-based practice, nurse leaders

## Abstract

**Introduction:**

Evidence-based practice (EBP) has been recognized as the gold standard for safe and high quality care. Nurse leaders have a strategic position in terms of initiating changes in clinical settings for successfully implementing EBP. Therefore, the factors that influence implementing EBP must be measured.

**Aims:**

To examine nurse leaders' attitudes, self-efficacy, and training needs for implementing evidence-based practice.

**Place and Duration of Study:**

Four teaching hospitals affiliated to Kerman University of Medical Sciences in the southeast of Iran from January to April 2014.

**Methods:**

A cross-sectional study was conducted on 70 nurse leaders from four teaching hospitals. After using a modified forward/backward translation procedure to create a Persian version of “perceptions of nurses of evidence-based practice questionnaire”, data were collected from the participants and analyzed using SPSS (version 20), descriptive statistics, Student's t-test, analysis of variance (ANOVA), and Pearson's correlation.

**Results:**

Most (82.86%) of the participants had not attended any specific training course on the implementation of EBP and 80% had not been involved in any research activities. Nurse leaders' attitudes toward EBP were unfavorable (mean=2.55±0.88), their levels of self-efficacy in EBP skills were weak (mean=2.64±1.31), and their demand for training in all of the EBP areas was moderate (3.89±.97).

**Conclusion:**

Current practice of nurse leaders is not evidence-based, which is worrisome and can result in serious deficiencies in the quality and safety of nursing care. Nurse leaders must attempt to equip themselves with the attitudes and skills required to change in practice using EBP.

## 1. Introduction

Rapid changes throughout the healthcare system have been followed by a greater emphasis on improving the quality of healthcare services, organizational performance, patient safety outcomes, and cost containment [1]. Therefore, it is the professional responsibility of nurses to apply the best scientific evidence for designing and implementing healthcare plans and integrate the accessible research evidence into their decision making [2,3]. In order to fulfill these responsibilities, evidence-based practice (EBP) was introduced as an important innovation in the healthcare system [4].

EBP is the deliberate use of available evidence in clinical decisions in combination with clinical expertise along with patients' concerns and preferences [5,6]. EBP is a problem-solving process [7] that consists of five sequential components,i.e.1) Formulating clinical answerable questions; 2) Seeking the most relevant evidence to the answers of these questions; 3) Critical appraisal of available evidence to determine its validity, relevance, and applicability; 4) Integrating and translating the research evidence and related knowledge into clinical practices; and 5) Reevaluating the appropriate application of the evidence and assessing the outcomes of various interventions [8,9].

In fact, it can be seen this central component is composed of a set of knowledge, attitudes, skills, and self-efficacy that nurses must have to implement EBP [10]. These competencies can elevate nurses' positions among multi-professional teams. Nurses who are involved in EBP have found a sense of professionalism and growth, which contributes to their professional identity and self-esteem [8]. The concept of self-efficacy is a “construct of Bandura's social cognitive theory, which is defined as a person's sense of confidence in her/his ability to perform or avoid a particular behavior in a variety of circumstances or settings” [6,7]. Self-efficacy is an individual mechanism and a predictor for understanding and influencing one's behavior in the context of EBP [11]. Lauder et al. reported that self-efficacy had positive impacts on academic motivation, learning, skill development, professional behavior, and job progress in nursing students [12].

Implementing EBP has potential benefits for healthcare providers, patients, and healthcare systems [8]. EBP reduces unsuitable variations in healthcare practices, supplies a set of standards for clinical decision making, enhances cost effectiveness, [13] and can lead to improve safety outcomes of patients by decreasing the clinical risks for clients and patients in line with the expectations of the patient and community from nursing practice. Therefore, EBP provides a framework for promoting excellence in healthcare and enhancing quality of clinical practice based on international standards [14].

Despite the expected benefits of EBP, various factors have made it difficult for nurses to integrate EBP into practice. Several researchers have reported that nurses have little knowledge of and an unfavorable attitude toward EBP. Many nurses do not recognize the concept of the term EBP or how to incorporate it into clinical practice [13,15-18]. Some other studies have reported that nurses have low self-efficacy in EBP activities. Nurses with higher levels of self-efficacy use research findings to a greater extent in clinical practice and also participate in the EBP implementation [1,7,9,19]. Researchers in another study showed that nurses' ability to implement EBP depends on several important factors, such as attitudes, understanding, knowledge, self-efficacy, and their leaders' support [20].

In Iran, the studies addressing EBP have mainly examined nurses' knowledge and attitudes toward EBP and the barriers to and facilitators of EBP [21-24]. We were unable to find a single comprehensive study investigating nurse leaders' attitudes, self-efficacy, and training needs in terms of EBP. In addition, it is obvious that the leadership behaviors of nurse' managers is also important in changing clinical settings and implementing EBP successfully [2]. Nurse leaders are responsible for ensuring that research findings are disseminated and providing the organizational support required to integrate these findings into clinical practices [25]. They have strategic positions in multi-disciplinary teams that would allow them to initiate changes in the healthcare system and to understand the importance of evidence-based interventions and policies [26]. For EBP to become widespread in reality and research results to be implemented and adapted in daily practice, the first necessary step would be the comprehensive assessment of the current situation. The results can help change the culture and environmental context in Iranian hospitals toward safe practice, thereby developing appropriate strategies to promote EBP in the healthcare system and to plan ways to reduce barriers, enhance knowledge, improve self-efficacy, revise the curriculum, and develop comprehensive and productive training programs.

The aims of this study were to examine 1- nurse leaders' attitudes toward EBP, 2- their self-efficacy, and 3- their training needs for adopting EBP.

## 2. Materials and methods

### 2.1 Research Design and Setting

This descriptive cross-sectional study was conducted at four teaching hospitals affiliated to Kerman University of Medical Sciences in the southeast of Iran from January to April 2014. Kerman is the largest city in the southeast of Iran and has the population of more than 722,000 people [27].

### 2.2 Sampling

The study's target population was all of the nurse leaders (N=95) that were employed at the time of the study. All the 95 nurse leaders including: head nurses, clinical supervisors, educational supervisors, and matrons [26] at the four hospitals were asked to participate, and 70 out of 95 nurse leaders (approximately 74%) completed the survey. The participants who had academic degrees in nursing and worked for more than one year as a nurse leader were considered eligible for the study.

### 2.3 Instrumentation and Data Collection

In this study, data were collected using “questionnaire of perceptions of nurses of evidence-based practice (EBP)”. This questionnaire was developed by a “team composed of faculty members from Nanyang Technological University and nursing representatives from Alexandra Hospital and National University in Singapore” [9], which was used with permission. In this study, for cross-cultural comparison of the translation [28-30], first, two researchers independently translated the original questionnaire accurately into Persian (forward translation); then, two independent translators who had no previous knowledge about the questionnaire and also were proficient in English language translated the text back into English (backward translation). The translated and original questionnaires were compared by three proficient people in both Persian and English and their agreement was assessed. Then, to ensure its face validity and assess nurses' understanding about the questions, the questionnaire was pilot-tested in the study settings by 25 nurses and five nurse leaders. These nurse leaders were excluded from participation in the study. According to their comments, some minor changes were made in the wording of the items. A group of experts, including eight faculty members of nursing and three medical informatics specialists, reviewed the draft for assessing qualitative content validity. Finally, the modified questionnaire was used. The reliability of each subsection of the original questionnaire was evaluated using Cronbach's alpha coefficient [19]. Therefore, the reliability of the questionnaire was evaluated using Cronbach's alpha coefficient (α=.89).

The questionnaire was composed of two sections. The first section was used to collect demographic information of the participants such as their gender, age, educational degree, job title, experience, professional training, and participation in activities such as evaluation of research reports, identification of researchable problems, participation in research, and utilization of research results. The second section was divided into three subsections, i.e.,1) Attitudes toward EBP (five items scored on a five-point Likert scale ranging from ‘Strongly disagree’ to ‘Strongly agree’); 2) Level of self-efficacy (nine items scored on a five-point Likert scale ranging from ‘Poor' to Excellent’); and 3) Training needs (seven items scored on a five-point Likert scale ranging from ‘Least important’ to ‘Most important’). After summing the scores and calculating their mean values, mean scores of above 4 and 3-4 as well as below 3 were considered as desirable, moderate, and unfavorable, respectively.

### 2.4 Ethical Consideration

All the processes and procedures of this study were approved by Ethics Committee and Research Council affiliated to Kerman University of Medical Sciences. The researchers also obtained permission from the directors of each hospital. The participants were briefed about the aims of the study and how to complete the questionnaire. They were assured that all of their information would remain confidential. Also, their informed consent forms were signed by the participants. No special ethical issue had occurred during the study's development and data collection.

### 2.5 Statistical Analysis

The data were analyzed by Statistical Package for Social Sciences (SPSS), version 20. Descriptive statistics was performed, including frequency, percentage, mean, and standard deviation (SD), and inferential statistics included independent samples *t*-test, analysis of variance (ANOVA), and Pearson's correlation. The level of significance was considered *P*≤ .05.

## 3. Results

### 3.1 Demographic Information

In this study, 74% response rate was obtained (70 out of 90 nurse leaders completed the survey). The majority of them were female (81.4%), aged between 35 and 45 years old (68.60%), had more than 10 years of nursing experience (82.86%), and had Bachelor's degree (87.14%). Most of the respondents were head nurses (51.43%), had shift rotation (51.40%), had not attended any specific training on the implementation of EBP in patient care (82.86%), and had not participated in any research activities, such as evaluation of research reports, identification of researchable problems, cooperative research, or use of research results in their clinical care during the past year (80%) (Table 1).

### 3.2 Attitudes toward EBP

The participants' attitudes toward EBP were unfavorable. Table 2 shows that the total mean scores of the attitudes of nurse leaders were 2.55±0.88.

The highest mean belonged to the statement “I prefer using more traditional methods instead of changing to new approaches” and the lowest mean score was related to the statement “My workload is too high to keep up-to-date with all new evidence.”

There were significant differences between the mean scores of the attitudes of participants based on length of experience (f=4.89, *p*=.02), age group (f=4.03, *p*=.01), and work shift (t=2.67, *p*=.03). No statistically significant differences were found between attitudes and attending EBP training as well as other demographic variables (*P*>.05).

### 3.3 Self-Efficacy of EBP Skills

Table 3 shows that the participants in this study perceived their levels of self-efficacy in EBP skills as weak (mean=2.64±1. 31). The highest mean score was for the item “Identify clinical problems” (3.38±1.06). The lowest mean score was for the item “Use a checklist to assess research articles” (2.37±1.35).

A significant difference was found between the mean scores of self-efficacy among the participants and age groups (f=5.53*, p*=.002), degree of nursing (t=2.44, *p*=.03), and attending training courses (t=3.25, *p*=.002). Pearson's correlation coefficient showed a moderately positive relationship between self-efficacy and attitudes of the nurse leaders (*r*=.46, *p*=.001), which indicated that the participants who had positive attitudes felt more confident and competent in EBP activities.

### 3.4 Training Needs

Total mean score indicated that the participants' demand for training was moderate (3.89±.97). The pattern of responses showed that the highest mean score of need was belonged to the statement “Identifying clinical issues for implementing EBP” (4.17±.93). Minimum mean score was related to the statement “Synthesizing evidence” (3.60±.99) (Table 4). Low positive correlation was observed between training needs and self-efficacy (r=.32, *p*=.006). There was no significant correlation between the perceived training needs and attitudes as well as demographic variables (*P*>.05).

## 4. Discussion

### 4.1 Principal Findings

The findings indicated that implementing and developing EBP are influenced by many factors. The nurse leaders were often working in challenging conditions and the majority of them had not attended any professional training on EBP and had not been involved in any research activities. Nurse leaders' attitudes toward EBP were unfavorable and levels of self-efficacy in EBP skills were not adequate. They scored receive training as moderate in almost all of the mentioned areas.

The findings also showed that most of the participants had not attended any professional training on EBP and had not participated in any research activities. The findings were largely similar to those of other studies [9,31]. Researchers in another study explained that, despite the fact that most nurses had an academic degree, they had not received any training in EBP as a part of their nursing education [31]. Nursing education in Iran is similar. The nursing curriculum in Iran lacks a systematic, focused, and uniform EBP education course. Thus, nursing graduates in Iran do not acquire pertinent information about EBP processes in healthcare. In addition, many of the graduates from M.Sc. and Ph.D. nursing programs in Iran tended to seek positions in educational institutions rather than clinical settings. Obviously, the lack of highly educated nurses in clinical settings is a problem in the pursuit of EBP [32].

The present results indicated that the participants had unfavorable attitudes toward EBP. They preferred using traditional methods over changing to new approaches. This reluctance to change might be explained by the current lack of experience/knowledge regarding nursing science, nursing research, and implementation of research results, all of which might be perceived as a threat to the existing, yet well-known, system. According to Breimaier et al. [33] nurses had unfavorable attitudes toward EBP, almost half of the nurses disagreed with changing nursing profession to a evidence-based profession, and 60% believed that research activities should not be considered in evaluating the performance of nurses and promotions to senior posts. Several studies in recent years have reported positive changes in both practitioners' and leaders' attitudes toward EBP [34,35]. This difference in the present result might be due to the reason that the existing organizational structure and culture have created barriers such as lack of time, unbalanced nurse– patient ratios, and lack of autonomy for changing practice of nurses [22]. Despite the complexity of organizational factors, such as culture, structures and processes, change in the organizational context is possible, but not easy. Thus, it is essential to identify potential strategies for change by nurse leaders [36].

One of the concerns of this study was the low self-efficacy levels expressed by the nurse leaders for engagement in EBP activities, which are not appropriate for EBP progress at all. Several studies have reported that self-efficacy is a modifiable factor that has a strong relationship with greater acceptance and use of all EBP activities [1,7,37] by ameliorating the inhibiting lack of confidence in EBP implementation [22,34].

These concerns are important, because nurse leaders are responsible for transforming the nurses' work environment and promoting safe and high quality care in clinical environments. Nurse leaders must organize the required resources for building the necessary infrastructures to support EBP [38]. They also may require additional knowledge and skills in generating and synthesizing, critically appraising research evidence [39] and translating them into practice. They should be role models and must be able to influence the promotion and implementation of EBP [2]. If nurse leaders do not believe in the value of EBP, its implementation will be impossible. Awareness of nurse leaders' attitudes and self-efficacy can be a criterion for assessing readiness for the EBP implementation process [4].

The association between self-efficacy of EBP skills and participants' attitudes supports the importance of attitudes in developing EBP process [4]. It indicates that participants had more positive attitudes and felt more confident and competent in their abilities to implement EBP activities. The relatively negative attitudes of the participants toward EBP in the present study were probably due to their low self-efficacy. These findings were consistent with those of other studies [4,40,41].

In the present work, significant differences were observed in the participants' attitudes and self-efficacy in terms of the variables such as years of experience, age group, and educational degree.Two systematic reviews have indicated that individual determinants influence nurses' use of research. The main determinants are attitudes and beliefs, contributions to research activities, education, attending conferences, seeking information behaviors, professional characteristics, job satisfaction, type of degree, completion of research classes, years since basic education, current position (for example, managerial and leadership positions) and other socio-economic factors [17,42].

In the present study, the participants stated high demand for training in all areas of EBP. They needed further training on the essential component of EBP and on methods to implement it in clinical practice, which was consistent with some previous studies [15,31,33]. Researchers reported that most nurses had willingness to share in training in terms of the fundamental principles of nursing research, application of research evidence in daily practice, and use of databases and libraries. They suggested that research-based needs assessment would generate an evidence-based framework for setting up training programs and undertaking organizational strategic planning [33]. Without this focus on educational planning, it is expected that nurses will not accept and implement EBP.

There was no difference in perceived training needs with demographic variables, nor were there any significant correlation between training needs and two variables of attitude and self-efficacy. These results were inconsistent with the results reported in an earlier research [43], which was probably due to the start of quality improvement programs such as clinical governance and accreditation and a strong emphasis on EBP in these programs; these issues caused all nurses to consider training useful and tend to receive additional training.

Lack of necessary awareness, positive attitudes, and self-efficacy by nurse leaders and nurses and having no evidence-based care plans can be the hindrance for providing safe care. Clearly, improving nurses' attitudes and skills in terms of implementing EBP plans enhances patients' safety outcome and quality of care. “For effective and safe care, nursing graduates need to attain the vital competencies set forth by Quality and Safety Education for Nurses (QSEN) initiative. Developing the QSEN initiative focuses on enhancing nursing curricula and fostering faculty development to support student achievement of quality and safety competencies” [44].

### 4.2 Limitations

This study had two limitations. First, the response rate of the nurse leaders was 74%, which may have had some influence on the significance of the results. Second, in this study, use of self-reporting questionnaires for assessing self-efficacy may have resulted in exaggerated scores. Self-report questioning could be subject to personal bias. Future studies can use a competency test to help determine the actual skills. For more reliable findings, a triangulation in data collection such as interviews and observation can be helpful as well.

## 5. Conclusion

The findings of this study pointed out that healthcare is not moving adequately toward high quality and safe care by EBP. Iranian nurse leaders use task-based approach, because they have little self-efficacy to integrate EBP into their practices and their attitudes toward EBP are unfavorable. Nurse leaders, as supporters and facilitators of EBP, should find ways to support and encourage nurses for change in practice. Therefore, nurse leaders' attitudes toward EBP and their behaviors influence staff's attitudes and behaviors. Nurses need and deserve knowledgeable leaders who are experts. Such leaders can provide a professional environment that will result in the personal and professional growth of the staff and finally quality improvement and safety of care. We suggest conducting further qualitative and quantitative studies to evaluate the effect of different approaches and enhance the EBP competencies of healthcare providers.

## Figures and Tables

**Table 1:** Demographic information (n=70)

Variables		N	%
**Sex**	Female	57	81.4
	Male	13	18.6

**Age groups**	<25	1	1.42
	25-35	7	10.0
	35-45	48	68.60
	>45	14	20.0

**Experience**	<5	2	2.85
	5 – 10	10	14.29
	>10	58	82.86

**Degree**	Bachelor	61	87.14
	Masters	9	12.86

**Job title**	Matron	2	2.86
	Educational Supervisor	2	2.86
	Clinical Supervisor	30	42.85
	Head Nurse	36	51.43

**Work shift**	Rotation	34	48.60
	Fixed	36	51.40

**Attending training of EBP**	Yes	12	17.14
	No	58	82.86

**Participate in research activities**	Yes	14	20.0
	No	56	80.0

**Table 2:** Attitudes toward EBP

Statement	Mean*	SD
1. I prefer using more traditional methods instead of changing based on new approaches	3.60	1.06
2. Most research articles are not relevant to my daily Practice	3.04	0.87
3. I believe evidence-based practice has only limited effectiveness	2.97	0.79
4. My clinical practices which are based on established methods, should not be questioned by people)	1.80	1.02
5. My workload is too high to keep up-to-date with all new evidences	1.34	0.65
Total	2.55	0.88

**Table 3:** Self-efficacy to undertake different activities EBP

Statement	Mean*	SD
**I am able to:**		
1. Identify clinical issues/problems.	3.38	1.06
2. Formulate a clinical issue/problem into a well-designed clinical question.	2.87	1.27
3. Distinguish between different types of questions (e.g., intervention, prognosis, harm, and cost-effectiveness).	2.68	1.31
4. Conduct online searches (using databases and web search engines).	2.54	1.36
**When reading research article, I am able to:**		
5. Relate research finding to my clinical practice and point out similarities and differences.	2.60	1.38
6. Use a check list to assess research articles.	2.37	1.35
7. Read a research report and have a general notion about its strength and weaknesses	2.38	1.33
**When applying research recommendations, I am able to:**		
8. Apply an intervention based on the most applicable evidence.	2.44	1.34
9. Evaluate the application of intervention and identify areas of improvement.	2.51	1.44
Total:	2. 64	1. 31

* Mean calculated from ratings on a 5-point scale; from 1 (Poor) to 5 (Excellent)

**Table 4:** Training needs

Statement	Mean	SD
1. Understanding what constitutes EBP	3.97	0.96
2. Identifying clinical issues for implementing EBP	4.17	0.93
3. Conducting literature searches	4.11	0.94
4. Conducting critical appraisal of articles	3.93	0.93
5. Synthesizing evidence	3.60	0.99
6. Implementing recommendations to practice	3.67	0.94
7. Understanding research methods and statistical terms	3.76	1.09
Total	3.89	0.97

Mean calculated from ratings on a 5-point scale; from 1 (Least important) to 5 (Most important)
